# TIGIT modulates sepsis-induced immune dysregulation in mice with preexisting malignancy

**DOI:** 10.1172/jci.insight.139823

**Published:** 2021-06-08

**Authors:** Wenxiao Zhang, Jerome C. Anyalebechi, Kimberly M. Ramonell, Ching-wen Chen, Jianfeng Xie, Zhe Liang, Deena B. Chihade, Shunsuke Otani, Craig M. Coopersmith, Mandy L. Ford

**Affiliations:** 1 Department of Surgery, Emory University School of Medicine, Atlanta, Georgia, USA.; 2 Department of Critical Care Medicine, People’s Hospital of Zhengzhou University (Henan Provincial People’s Hospital), Zhengzhou, China.; 3 Department of Critical Care Medicine, Zhongda Hospital, Southeast University, Nanjing, China.; 4 Department of General Medical Science, Chiba University Graduate School of Medicine, Chiba, Japan.; 5 Department of Emergency and Critical Care Medicine, Eastern Chiba Medical Center, Togane, Japan.; 6 Emory Critical Care Center and; 7 Emory Transplant Center, Emory University School of Medicine, Atlanta, Georgia, USA.

**Keywords:** Immunology, Inflammation, Bacterial infections, Cancer, T cells

## Abstract

TIGIT is a recently identified coinhibitory receptor that is upregulated in the setting of cancer and functionally contributes to the impairment of antitumor immunity. However, its role during sepsis is unknown. Because patients with cancer are 10 times more likely to die of sepsis than previously healthy (PH) patients with sepsis, we interrogated the role of TIGIT during sepsis in the context of preexistent malignancy. PH mice or cancer (CA) mice inoculated with lung carcinoma cells were made septic by cecal ligation and puncture (CLP). We found that sepsis induced TIGIT upregulation predominantly on Tregs and NK cells in both PH and CA mice. Anti-TIGIT Ab improved the 7-d survival of CA septic mice but not PH mice after CLP. Treatment of CA septic animals but not PH septic animals with anti-TIGIT mAb significantly reversed sepsis-induced loss of CD4^+^ T cells, CD8^+^ T cells, Foxp3^+^ Treg, and CD19^+^ B cells in the spleen, which was the result of decreased caspase-3^+^ apoptotic cells. In sum, we found that anti-TIGIT Ab reversed sepsis-induced T cell apoptosis in CA septic mice and led to a significant survival benefit, suggesting its use as a potential immunotherapy to improve outcomes in septic patients with cancer.

## Introduction

Sepsis is a life-threatening condition of multiorgan dysfunction resulting from a dysregulated host response to infection (1). Despite intense efforts, sepsis remains a critical problem with substantial morbidity and mortality. This is evidenced by results from a 2020 *Lancet* study demonstrating that approximately 20% of the world’s population dies from complications of sepsis (2), and that sepsis-related healthcare costs amount to approximately $60 billion per year in the United States alone (3). Additionally, other than antibiotics and supportive care, there is no clinically effective therapy for sepsis (4).

Accumulating evidence has revealed that immunosuppression plays a pivotal role in the progress and prognosis of sepsis (5). This immunosuppressive state manifests as a decrease in both the number and functionality of immune cells and the upregulation of T cell coinhibitory receptors (6). Seminal studies targeting programmed cell death 1 (PD-1) (7) and cytotoxic T lymphocyte antigen 4 (CTLA-4) (8) have shown promising results in mitigating immune dysregulation and mortality in experimental sepsis models of sepsis, and clinical studies targeting PD-1 and PD-L1 have shown promise in human septic patients (9, 10). Importantly, immunotherapies directed against these coinhibitory receptors have exhibited unprecedented benefit in clinical trials for multiple types of cancer (11).

T cell Ig and ITIM domain (TIGIT), belonging to a poliovirus receptor family of type 1 proteins, is a novel coinhibitory receptor. TIGIT is expressed on peripheral memory and regulatory CD4^+^ T cells and NK cells and is upregulated on naive CD4^+^ T cells after their activation. Parallel to the relationship between CD28 and CTLA-4, TIGIT and CD226 compete for ligands (CD155 and CD112) (12). After CD155 or CD112 binds to TIGIT, a negative signaling event is triggered through the 2 ITIM domains on its cytoplasmic tail (13). Thus, TIGIT has been shown play a protective role in autoimmunity, and it negatively regulates the immune response to cancer by inducing T cell exhaustion (14, 15).

Many septic patients have comorbidities, and cancer is the most common (16). Cancer leads to a higher risk of developing sepsis (17) and significantly increases ICU and in-hospital mortality rates compared with septic patients without preexisting malignancy (18, 19). Although the etiology behind the increased mortality seen in septic patients with cancer is likely multifactorial (20), our previous studies (21, 22) in mouse models indicate that cancer septic animals have increased mortality even in the absence of any chemotherapy or other cancer treatment. This suggests that the presence of chronic inflammation as a result of cancer may influence systemic immunity. However, the role of the TIGIT pathway in the observed increased sepsis mortality in animals with preexisting malignancy is unknown. Here, we aimed to study the role of TIGIT signaling during sepsis in both PH mice and mice with preexisting malignancy. Results indicate that anti-TIGIT treatment mitigated lymphocyte depletion and sepsis mortality in cancer (CA) septic mice but not in previously healthy (PH) mice, illuminating a potential therapeutic target for septic patients with preexisting malignancy.

## Results

### TIGIT was upregulated on T cells during sepsis in both PH mice and mice with preexisting CA.

**Figure 1:**
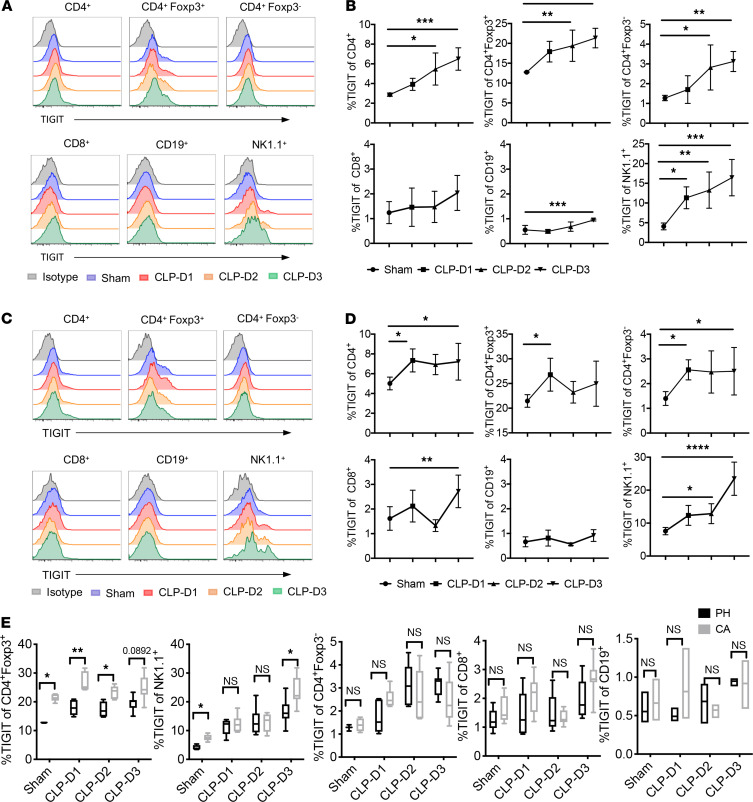
TIGIT is upregulated on splenic lymphocytes isolated from PH septic mice and septic mice with preexisting malignancy (CA). B6 mice were injected with LLC and monitored for 3 weeks. PH and CA mice were subjected to CLP (*n* = 22/group) or sham surgery (*n* = 5/group). Mice were sacrificed on days 1, 2, and 3 after CLP and TIGIT expression on splenic immune cells was measured. (**A** and **C**) Representative flow histograms showing TIGIT expression on the indicated lymphocyte populations isolated from either PH or CA septic mice. Plots were gated on CD4^+^, CD4^+^Foxp3^+/–^, CD8^+^, NK1.1^+^, and CD19^+^ cells, respectively. (**B** and **D**) Summary data of the percentage of TIGIT^+^ lymphocytes isolated from either PH or CA septic mice. (**E**) TIGIT expression on each cell subset was compared between PH and CA mice at different time points. Groups were compared with 2-way ANOVA with Tukey’s post hoc test. *P ≤ 0.05, **P ≤ 0.01, ***P ≤ 0.001. PH, previously healthy; CA, cancer; LLC, Lewis lung carcinoma; CLP, cecal ligation and puncture; TIGIT, T cell Ig and ITIM domain.

It has been previously demonstrated that TIGIT is expressed on memory T cells, Tregs, and NK cells and becomes upregulated upon stimulation (9). However, the kinetics and distribution of TIGIT expression during sepsis is not known. Therefore, we first examined the kinetics of TIGIT expression on different lymphocyte lineages after a septic insult in both PH and CA animals. In PH mice, TIGIT was upregulated on total CD4^+^ T cells, Foxp3^+^ Treg, CD4^+^ Foxp3^–^ T conventional cells (Tconvs), and NK cells on days 2 and 3 after sepsis compared with sham controls (Figure 1, A and B). Although TIGIT was upregulated on B cells by day 3 after cecal ligation and puncture (CLP), overall expression was negligible compared with other cell populations. TIGIT was also upregulated on total CD4^+^ T cells, Foxp3^+^ Treg, CD4^+^ Foxp3^–^ Tconv, and NK cells after sepsis in CA mice (Figure 1, C and D). In addition, TIGIT expression on CD8^+^ T cells in CA septic mice increased significantly by day 3 compared with CA sham controls. Comparisons between PH and CA septic mice indicated that CA mice expressed significantly higher levels of TIGIT on Foxp3^+^ Treg and NK cells than PH mice prior to the septic insult, and this difference persisted after CLP (Figure 1E). Taken together, these data reveal that TIGIT was most prominently upregulated on Treg and NK cells after CLP in both PH and CA mice, but that the cell type that exhibited the greatest differential TIGIT expression between PH and CA mice was Foxp3^+^ Treg.

### TIGIT expression defined a population of CTLA4^hi^ Helios^hi^ PD-1^hi^ Treg during sepsis.

**Figure 2:**
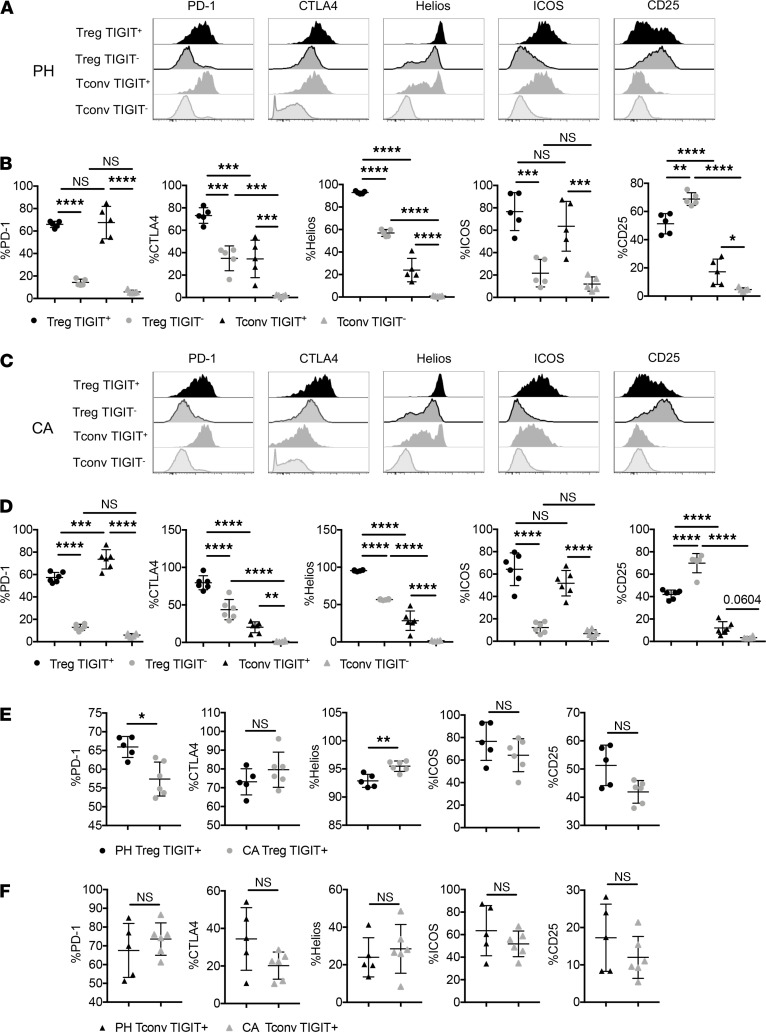
TIGIT is coexpressed with other coinhibitory receptors and activating markers. CA and PH mice were subjected to CLP and spleens were harvested 1 d after CLP. (**A** and **C**) Representative flow cytometric histograms for PD-1, CTLA-4, Helios, ICOS, and CD25 expression on TIGIT^+^ and TIGIT^–^ CD4^+^ T cells in CA and PH CLP mice, respectively. (**B** and **D**) Summary data of the frequencies of PD-1^+^, CTLA-4^+^, Helios^+^, ICOS^+^, and CD25^+^ cells within TIGIT^+^ and TIGIT^–^ CD4^+^ T cells in CA and PH CLP mice, respectively. (**E** and **F**) PD-1, CTLA-4, Helios, ICOS, and CD25 expression on TIGIT^+^ Treg and TIGIT^+^ Tconv were compared between PH and CA septic mice. (**A–D**) Groups were compared with 2-way ANOVA with Tukey’s post hoc test. (**E** and **F**) Groups were compared using Mann-Whitney *U* test. *P ≤ 0.05, **P ≤ 0.01, ***P ≤ 0.001, ****P < 0.0001. PH, previously healthy; CA, cancer; CLP, cecal ligation and puncture; TIGIT, T cell Ig and ITIM domain; PD-1, programmed cell death 1; CTLA-4, cytotoxic T lymphocyte antigen 4; Tconv, T conventional cell.

A dysregulated T cell response can involve the coexpression of coinhibitory receptors, an accumulation of Tregs, and deficits in effector cell functions. Therefore, we examined activation and coinhibitory receptor expression on TIGIT^+^ Treg and TIGIT^+^ Tconvs in both PH and CA septic animals. As shown in Figure 2, A–D, TIGIT^+^ Treg contained a higher frequency of PD-1^+^, CTLA-4^+^, Helios^+^, and ICOS^+^ cells in both PH and CA septic mice. In contrast, CD25 expression was significantly lower on TIGIT^+^ Treg than TIGIT^–^ Treg in both PH and CA mice. Taken together, these phenotypic profiles suggest that in both PH and CA septic mice, TIGIT^+^ Treg had a PD-1^+^ CTLA-4^+^ ICOS^+^ Helios^+^ phenotype, which is consistent with more highly suppressive Treg, as shown in multiple recent reports (23–29). Direct comparison between TIGIT^+^ Treg in PH versus CA septic mice revealed that TIGIT^+^ Treg in PH mice exhibited significantly higher expression of PD-1 and lower expression of Helios, one of the Ikaros family transcription factors associated with Treg stability (Figure 2E). There were no differences in expression of these molecules between TIGIT^+^ Tconv isolated from PH versus CA septic animals (Figure 2F).

### TIGIT^+^ T cells isolated from septic hosts exhibited distinct patterns of cytokine production.

**Figure 3:**
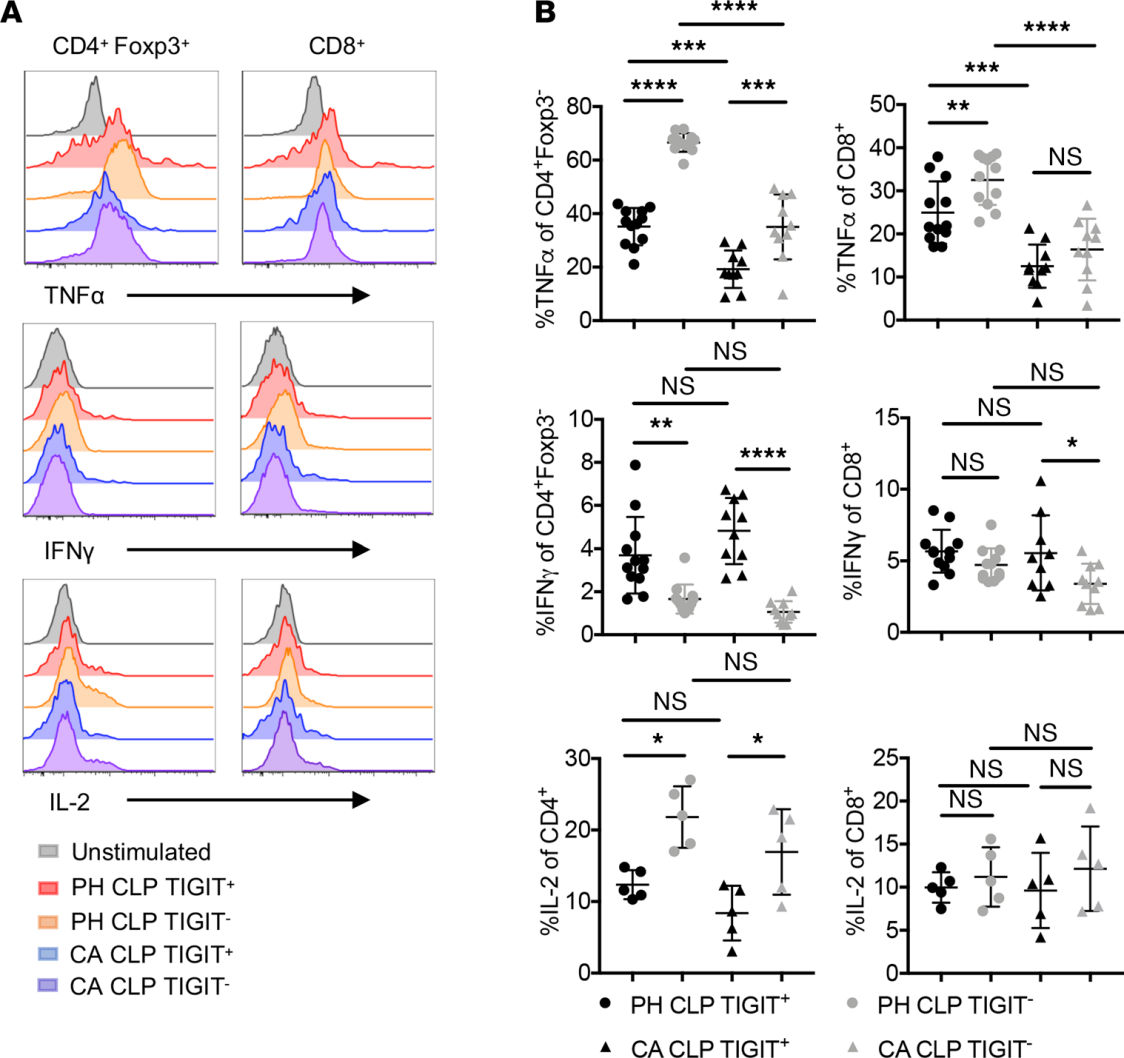
TIGIT^+^ and TIGIT^–^ T cells isolated from septic hosts exhibit distinct patterns of cytokine production. Splenocytes from CA and PH septic mice were harvested 1 d after CLP and stimulated with PMA and ionomycin at 37°C for 4 h. Data are derived from 2 independent experiments. (**A**) Representative flow cytometry graphs of TNF-α, IFN-γ, and IL-2 expression on CD4^+^ Tconv and CD8^+^ T cells. (**B**) Summary data of TNF-α^+^, IL-2^+^, and IFN-γ^+^ cells in CD4^+^ and CD8^+^ T cells. Groups were compared with 2-way ANOVA with Tukey’s post hoc test. *P ≤ 0.05, **P ≤ 0.01, ***P ≤ 0.001, ****P < 0.0001. PH, previously healthy; CA, cancer; CLP, cecal ligation and puncture; TIGIT, T cell Ig and ITIM domain; Tconv, T conventional cell.

We next examined whether TIGIT^+^ CD4^+^ and TIGIT^+^ CD8^+^ T cells in CA septic mice exhibited defects in effector function. After stimulating single-cell suspensions of splenocytes with PMA and ionomycin, we found that TIGIT^+^ CD4^+^ T cells were poor producers of IL-2 and TNF-α compared with TIGIT^–^ CD4^+^ T cells but produced much higher levels of IFN-γ (Figure 3, A and B). We also noted a significant increase in TNF production in both TIGIT^+^ and TIGIT^–^ CD4^+^ cell subsets in PH versus CA mice (Figure 3B). CD8^+^ T cells that were TIGIT^+^ produced less TNF in PH mice but not CA mice (Figure 3B). However, CA TIGIT^+^ CD8^+^ T cells produced more IFN-γ than TIGIT^–^ cells, whereas PH CD8 T cells generated a similar level of IFN-γ. There was no difference in IL-2 production between TIGIT^+^ CD8^+^ T cells and TIGIT^–^ CD8^+^ T in either PH or CA mice. Taken together, these data demonstrate that TIGIT expression was associated with deficits in T effector (Teff) cell function.

### Anti-TIGIT mAb treatment improved survival in CA septic mice but not PH septic mice.

**Figure 4:**
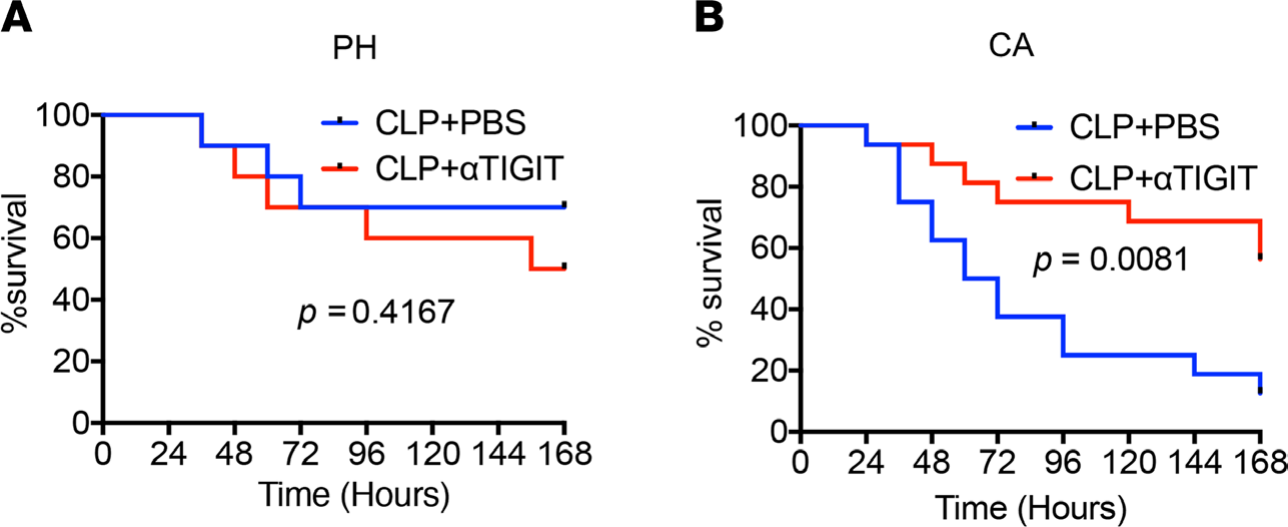
Anti-TIGIT mAb treatment improved survival in CA septic mice but not PH septic mice. PH and CA septic mice were treated with anti-TIGIT mAb or PBS as a control. Mice were observed every 12 h and 7-d survival was monitored. (**A**) Seven-d survival in PH septic mice (*n* = 16/group, *P* = ns). (**B**) Seven-d survival in CA septic mice (*n* = 10/group, *P* = 0.0081). Kaplan-Meier survival curves were compared using the log-rank test. PH, previously healthy; CA, cancer; CLP, cecal ligation and puncture; TIGIT, T cell Ig and ITIM domain.

PH and CA mice were subjected to CLP and treated with either an anti-TIGIT mAb at 1 h and 12 h after CLP or the same volume of PBS as a vehicle control (in some experiments, isotype control was also used and yielded results identical to PBS control). This timing of administration was chosen because expression of TIGIT on CD4^+^ T cells and Foxp3^+^ Treg began to increase around 24 h after CLP (Figure 1). Thus, we rationalized that the Ab treatment should be “onboard” during this initial increase and chose to initiate Ab treatment prior to that at 12 h after CLP. Results indicated that whereas anti-TIGIT mAb failed to improve sepsis survival in PH septic mice, the survival of CA septic mice was significantly improved by anti-TIGIT (Figure 4, A and B). Of note, this improved mortality was not associated with altered inflammatory or antiinflammatory cytokine profile (IL-1β, IL-6, TNF, MCP-1, IFN-γ, or IL-10; Supplemental Figure 1; supplemental material available online with this article; https://doi.org/10.1172/jci.insight.139823DS1) or altered serum concentration or organ damage markers such as ALT, AST, or creatinine (Supplemental Figure 2). Moreover, it was not associated with improved tumor clearance because there was no effect of anti-TIGIT on tumor size during the 7-d observation period (not shown). Of note, we performed the same basic experiment but instead using CA mice exposed to endotoxin (LPS) and did not observe improved survival in the anti-TIGIT–treated mice relative to control-treated mice (Supplemental Figure 3). These findings corroborate the conclusion that the mortality difference in anti-TIGIT–treated CA mice in the CLP model is not mediated via the magnitude of the inflammatory response. Thus, taken together, these data indicate that the therapeutic targeting of the TIGIT pathway was effective in septic animals with preexisting malignancy but not in PH septic animals.

### Anti-TIGIT mAb administration reverses lymphopenia in CA septic mice but not PH septic mice.

**Figure 5:**
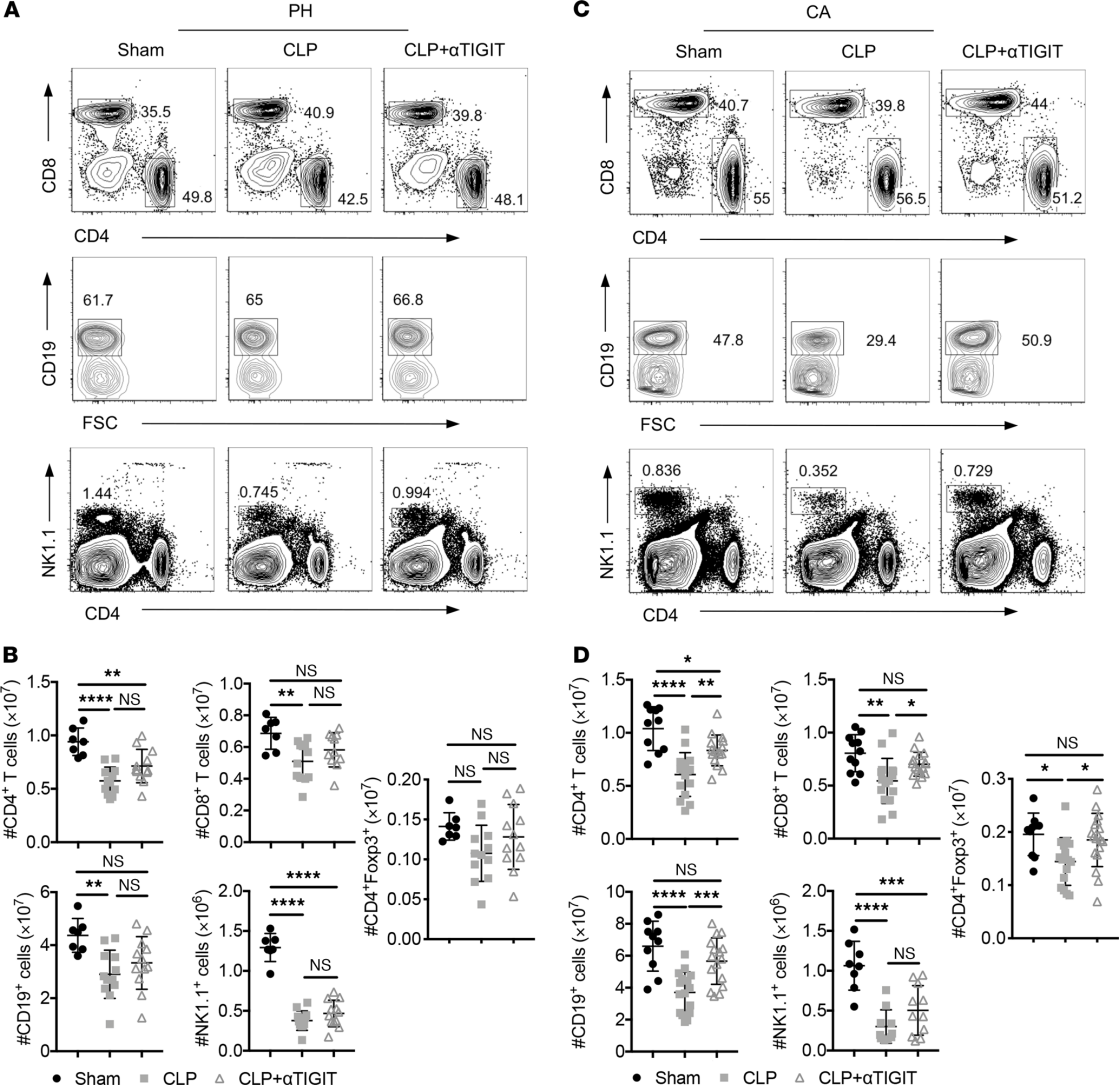
Anti-TIGIT mAb administration reversed lymphopenia in CA septic mice but not PH septic mice. PH and CA septic mice were treated with either anti-TIGIT mAb or the same volume of PBS (*n* = 7 mice/group) as described above. Mice were subjected to sham surgery as a control (*n* = 5 mice/group). Animals were sacrificed, and spleens were harvested 2 d after CLP. Data were derived from 2 independent experiments. (**A** and **B**) Representative flow cytometry plots of lymphocyte gating and summary data of lymphocyte numbers from PH mice. (**C** and **D**) Representative flow cytometry plots of lymphocyte gating and summary data of lymphocyte numbers from CA mice. Groups were compared with 2-way ANOVA with Tukey’s post hoc test. *P ≤ 0.05, **P ≤ 0.01, ***P ≤ 0.001, ****P ≤ 0.0001. PH, previously healthy; CA, cancer; CLP, cecal ligation and puncture; TIGIT, T cell Ig and ITIM domain.

We next interrogated the cellular mechanisms underlying the ability of the anti-TIGIT treatment to protect CA septic mice from death during sepsis but not PH septic mice. Sepsis caused prominent cell loss in the CD4^+^ and CD8^+^ T cell, NK cell, and B cell compartments in PH mice (Figure 5, A and B) and CA septic mice (Figure 5, C and D). The loss of CD4^+^ T cell, CD8^+^ T cell, Foxp3^+^ CD4^+^ Treg, NK cell, and B cell compartments was unchanged after anti-TIGIT in PH septic mice (Figure 5C). In contrast, the CD4^+^ and CD8^+^ T cell and B cell compartments in CA septic mice were increased after anti-TIGIT administration (Figure 5D). Importantly, anti-TIGIT also increased the number of Foxp3^+^ CD4^+^ Treg in CA septic animals (Figure 5D). Anti-TIGIT failed to rescue NK cell loss in either PH septic or CA septic animals.

### Anti-TIGIT mAb decreased T cell expression of PD-1 in CA septic but not PH septic mice.

**Figure 6:**
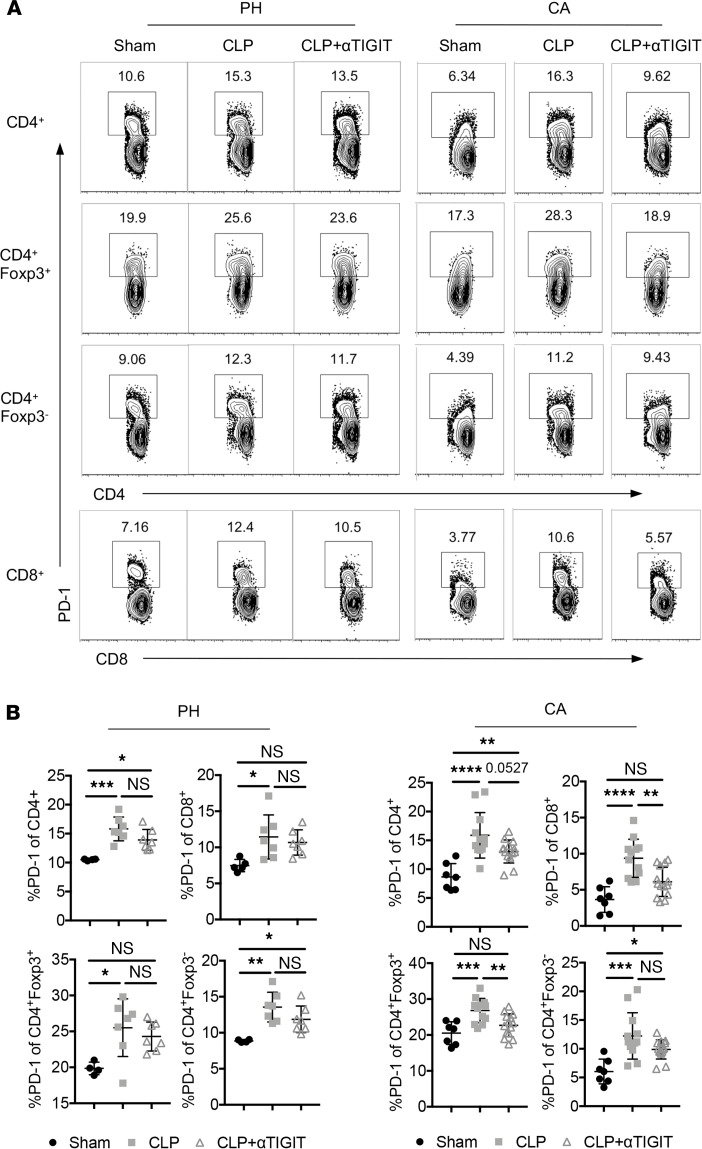
Anti-TIGIT mAb resulted in decreased expression of PD-1 on T cells in CA septic mice but not PH septic mice. PH and CA mice were subjected to CLP and either treated with anti-TIGIT mAb or the same volume of PBS (*n* = 7 mice/group) as described above. Mice were subjected to sham surgery as a control (*n* = 5). Animals were sacrificed, and spleens were harvested 48 h after CLP. Data were derived from 2 independent experiments. (**A**) Representative flow cytometry plots of PD-1 expression on T cells in sham, CLP and CLP plus αTIGIT groups with or without preexistent malignancy. (**B**) Data summary of PD-1 percentages on CD4^+^ T cells, CD8^+^ T cells, Treg, and Tconv cells from both PH and CA mice. Groups were compared with 2-way ANOVA with Tukey’s post hoc test. *P ≤ 0.05, **P ≤ 0.01, ***P ≤ 0.001, ****P ≤ 0.0001. PH, previously healthy; CA, cancer; CLP, cecal ligation and puncture; TIGIT, T cell Ig and ITIM domain; PD-1, programmed cell death 1; Tconv, T conventional cell.

We then examined the impact of anti-TIGIT treatment on the expression of other coinhibitory receptors. Anti-TIGIT had no impact on PD-1 expression on CD4^+^ or CD8^+^ Tconv and Treg in PH septic mice (Figure 6, A and B). However, anti-TIGIT treatment resulted in a reduction in the frequency of PD-1^+^ cells within the CD4^+^, CD8^+^, and Foxp3^+^ Treg compartments of CA septic mice (Figure 6, A and B). Anti-TIGIT did not impact TNF, IFN-γ, or IL-2 production by either CD4^+^ (Foxp3^+^ or Foxp3^–^) or CD8^+^ T cells in either PH (not shown) or CA septic animals (Supplemental Figure 4). In addition, anti-TIGIT mAb did not alter the expression of CD25 and CTLA-4 in either Foxp3^+^ CD4^+^ cells or Foxp3^–^ CD4^+^ cells in either PH septic (not shown) or CA septic hosts (Supplemental Figure 5). Collectively, these data reveal that anti-TIGIT treatment during CA sepsis was associated with a reversal of immune cell loss and a decrease in the frequency of PD-1^+^ Treg and Teff in hosts with preexisting malignancy.

### Anti-TIGIT mAb administration inhibited lymphocyte apoptosis in CA septic mice.

**Figure 7:**
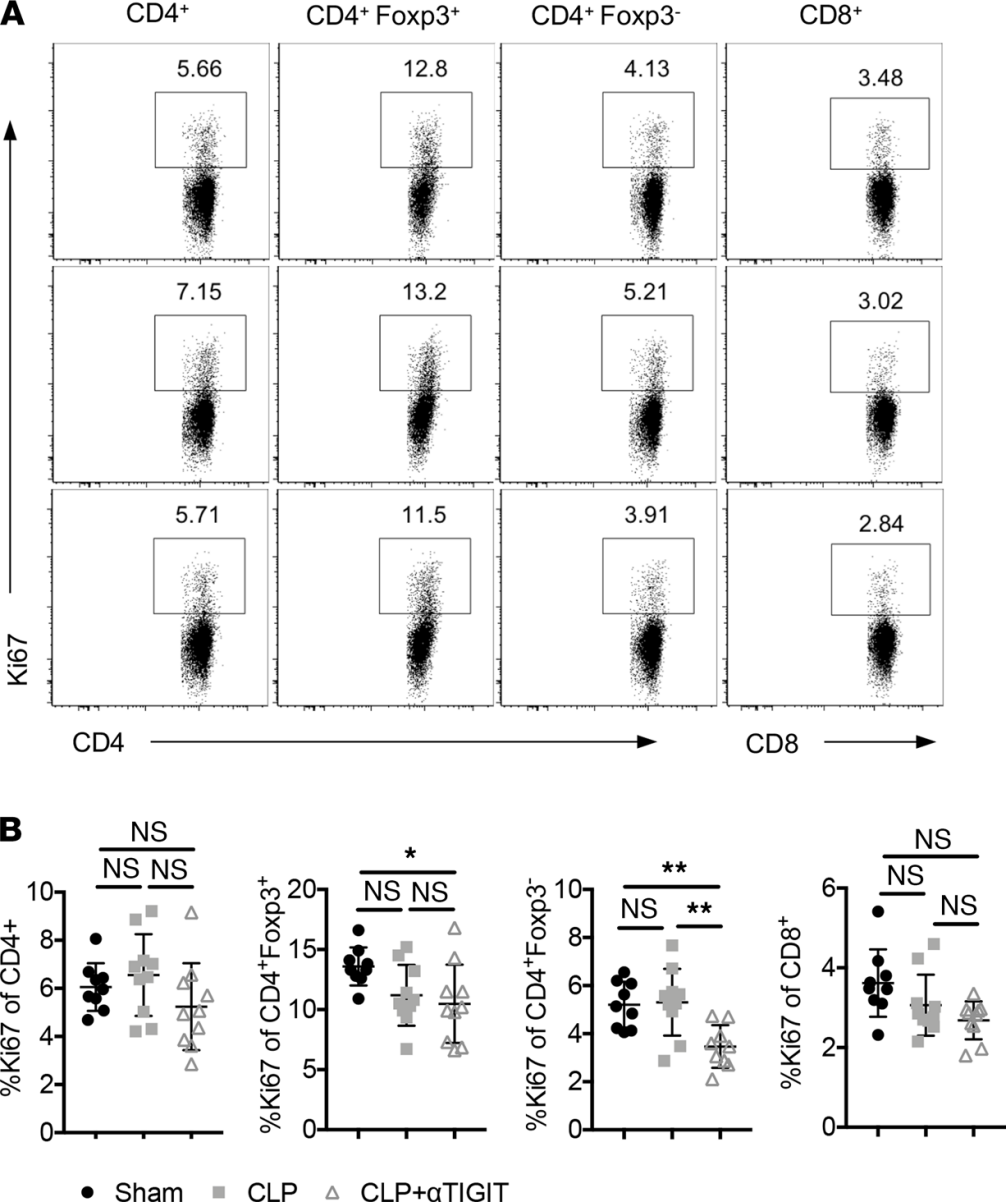
Decreased lymphocyte loss in anti-TIGIT–treated CA septic mice was not associated with increased proliferation. CA mice were subjected to CLP and either treated with anti-TIGIT mAb or the same volume of PBS as described above. Mice were subjected to sham surgery as a control. Spleens were collected for intracellular Ki67 staining. Data were derived from 2 independent experiments. *n* = 9–10/group. (**A**) Representative flow cytometry plots of Ki67 expression on total CD4^+^ T cells, Treg, Tconv, and total CD8^+^ T cells in the 3 groups. (**B**) Data summary of Ki67 percentages on different cell subsets. Groups were compared with 2-way ANOVA with Tukey’s post hoc test. *P ≤ 0.05, **P ≤ 0.01. CA, cancer; CLP, cecal ligation and puncture; TIGIT, T cell Ig and ITIM domain; Tconv, T conventional cell.

Alterations in the number of immune cells may be the result of changes in cell proliferation, increased cell death, or cell redistribution. To determine whether anti-TIGIT impacted cell proliferation in the setting of CA sepsis, we measured the frequency of Ki-67^+^–proliferating T cells in the spleens of anti-TIGIT–treated CA septic mice versus PBS-treated CA septic mice. Anti-TIGIT treatment did not increase the proportion of Ki-67^+^–proliferating cells among CD4^+^ or CD8^+^ Tconv or Foxp3^+^ Treg (Figure 7, A and B). These data indicate that the observed increase in T cell numbers after anti-TIGIT administration was not the result of enhanced proliferation.

**Figure 8:**
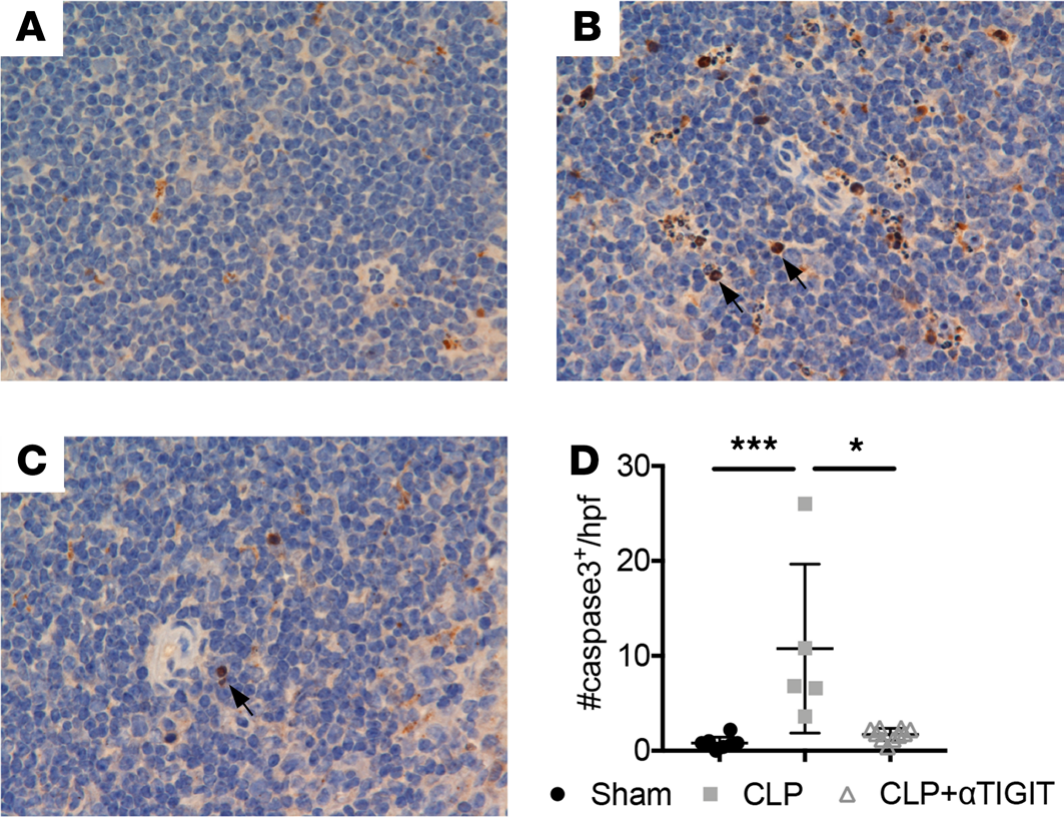
Anti-TIGIT mAb results in decreased splenic lymphocyte apoptosis in cancer septic mice. CA mice were subjected to CLP and either treated with anti-TIGIT mAb or the same volume of PBS as described previously. Mice were subjected to sham surgery as a control. (**A–C**) Representative immunohistochemistry images of caspase-3 staining in spleens isolated from different groups. Cell death was quantified in the spleen by counting cells staining positive for active caspase-3 (brown chromogen) as indicated by arrow in 5 random, high-powered (×400) fields. (**D**) Data summary of caspase-3 positive cell numbers in the 3 groups. (**A**: sham; **B**: CLP; **C**: CLP plus αTIGIT.) Data were derived from 2 independent experiments. Groups were compared with 2-way ANOVA with Tukey’s post hoc test. *P ≤ 0.05, ***P ≤ 0.001. CA, cancer; CLP, cecal ligation and puncture; TIGIT, T cell Ig and ITIM domain; 10x ocular x 40x objective.

Apoptosis is believed to play an essential role in sepsis-induced cell death. As such, we used IHC to evaluate apoptosis in the spleen 24 h after sepsis as measured by the detection of active caspase-3. As shown in Figure 8, A–D, there was extensive apoptosis in the spleen of CA septic mice compared with CA mice that underwent sham surgery. Anti-TIGIT administration significantly (*P* < 0.05) decreased the numbers of active caspase-3^+^ apoptotic cells per high-powered field. Overall, these data suggest that the protective effect of anti-TIGIT was attributed to a reduction in lymphocyte apoptosis in CA septic mice.

## Discussion

The development of checkpoint inhibitors has become the forefront of immune oncology, raising the possibility that these immunotherapeutics could be used in other immune-related diseases. Previous studies have elaborately explored the role of PD-L1/PD1 interactions in sepsis and show promising results in preliminary experiments and in ongoing clinical trials (7, 30). The complex immune dysregulation that exists during sepsis illuminates the need to explore other checkpoint molecules as potential therapeutic targets. In the current study, we found that sepsis induced TIGIT upregulation on immune cells both in PH mice and in the setting of preexisting malignancy. Further characterization of TIGIT-expressing cells revealed coexpression with other important coinhibitory receptors and activation markers, as well as defective cytokine secretion. Sepsis survival was significantly (*P* = 0.0081, Figure 4) improved in anti-TIGIT–treated mice with preexistent malignancy but not in PH mice. Finally, the protective effect observed in CA septic mice was associated with a decrease in apoptotic splenic cells.

Over the past few years, TIGIT has emerged as an important coinhibitory receptor and has been extensively studied in cancer, chronic infection, and autoimmune diseases (14, 31). Coinhibitory receptors can deliver signals directly to the T cell by recruiting phosphatases to their intracellular domains. By administering an agonistic anti-TIGIT Ab in the absence of antigen-presenting cells in vitro, Joller et al. (32) demonstrated functional inhibition in purified WT but not TIGIT^–/–^ T cells after stimulation with anti-TIGIT, indicating that TIGIT can act directly on T cells in a cell-autonomous fashion. Indeed, TIGIT contains 2 ITIMs in its cytoplasmic tail, which have been shown to mediate recruitment of the phosphatase SHIP-1 (12), thus providing a mechanism by which TIGIT can act cell intrinsically to dampen activating signals.

Although TIGIT is not appreciably expressed on B cells, in the current study we found that sepsis-induced B cell loss was also reversed by anti-TIGIT treatment, indicating that the treatment did not function solely in a cell-autonomous manner. The mechanism by which anti-TIGIT modulates B cell survival during sepsis remains to be elucidated. Further, the fact that anti-TIGIT did not result in significantly improved Teff cell cytokine function (Supplemental Figure 3) may be reflective of compensation by other coinhibitory receptors that are functionally redundant with TIGIT. Alternatively, it may indicate that cytokine effector function, unlike apoptosis, is not modulated by TIGIT signaling. We have also shown that anti-TIGIT treatment decreased PD-1 expression on T cells in CA septic mice. However, Chauvin et al. (15) observed that TIGIT blockade did not significantly impact PD-1 expression on antigen-specific CD8^+^ T cells in PBMCs from patients with melanoma. Differences in the model systems used may explain this discrepancy.

In addition to cell-autonomous effects, TIGIT has been found to inhibit T cell responses indirectly by delivering inhibitory signal to the CD155 ligand-expressing cells, thereby preventing DC maturation and inducing production of immunosuppressive cytokines (12). Furthermore, TIGIT marks a subset of Treg that exhibit higher expression of known Treg effector molecules and heightened suppressive capacity relative to TIGIT^–^ Treg (33). Although TIGIT is uniquely enriched on both tumor-infiltrating CD8^+^ T cells and Foxp3^+^ tumor tissue Treg, Kurtulus et al. demonstrated that it is the function of TIGIT on Treg that plays a critical role in dampening antitumor immune responses, rather than CD8^+^ Teff cells (34). Ligation of TIGIT on Treg cells induced expression of the effector molecule fibrinogen-like protein 2, which promoted Treg-mediated suppression of Teff cell proliferation (35). Consistent with previous studies (33, 34), we observed that TIGIT was highly expressed on Foxp3^+^ Treg and coexpressed with PD-1, CTLA-4, and Helios, which are associated with Treg suppressive function and stability.

The effect of TIGIT on T cell apoptosis has not been well established. One study observed no significant differences in the frequency of early or late apoptotic cells among human CD4^+^ T cells activated with anti-CD3/anti-CD28 and incubated with agonistic anti-TIGIT Ab (36). In the current study, anti-TIGIT Ab downregulated active caspase-3 (an important terminal molecule in the apoptosis pathway) on T cells in vivo after CLP. The antiapoptotic effect of the TIGIT mAb could be a result of the concurrent downregulation of PD-1 on T cells during sepsis. This notion is supported by previous studies showing that PD-1 signaling renders T cells susceptible to apoptosis (37). During sepsis, anti–PD-1 mAb markedly increases the expression of the antiapoptotic molecule Bcl-xL and results in improved survival (7). Interestingly, we found that treatment with anti-TIGIT mAb inhibited the apoptosis pathway, was associated with the downregulation of PD-1 expression, and resulted in improved survival in septic mice with preexistent malignancy but not in PH septic mice. This finding of PD-1 downregulation after anti-TIGIT is of particular interest because we recently reported that anti–PD-1 alone failed to improve sepsis mortality in hosts with preexisting malignancy (38). It is therefore interesting to speculate that anti-TIGIT and anti–PD-1 coblockade might be particularly effective at inhibiting sepsis mortality in animals with preexisting malignancy (31).

Previous studies have demonstrated that TIGIT competes for ligand binding with CD226, a costimulatory molecule important in antiviral and antitumor responses (36). TIGIT also directly blocks the homodimerization of CD226, physically preventing CD226 from signaling (31). Thus, in the current study, we cannot exclude the possibility that the observed effects were due to an indirect effect on CD226 signaling.

We used a sepsis model in which mortality in healthy B6 mice was approximately 50%. It is noteworthy that despite the same ligation length and puncture size, CA septic mice were weaker and experienced higher mortality than PH mice. Importantly, there were no changes in tumor size observed in the CA septic mice treated with anti-TIGIT mAb; these findings are consistent with a previous study in which anti-TIGIT treatment alone did not impact tumor size over a prolonged period of time (31). Although it is true that the CLP model may not recapitulate all etiologies of sepsis in humans, CLP as a model of ruptured appendicitis has been shown by a number of different labs to faithfully recapitulate the cytokine response observed in septic patients (39, 40). Our results showed that the same anti-TIGIT experiment using CA mice exposed to endotoxin (LPS) and did not result in improved survival in the anti-TIGIT–treated mice relative to control-treated mice. However, many studies have shown that LPS poorly recapitulates the immune derangements observed in clinical sepsis (39, 40). Thus, future investigation will be required to determine whether the effects of anti-TIGIT Ab identified in this Lewis lung carcinoma (LLC)/CLP model hold true for other models of sepsis and for other tumor types.

In summary, our study found that TIGIT was upregulated on Treg and NK cells in both PH septic mice and CA septic mice. Treatment targeting TIGIT improved the 7-d survival of CA septic mice but not PH septic mice. The improvement in sepsis survival was associated with a reduction in T cell apoptosis and in the coexpression of PD-1 on the Treg and Teff CA septic mice. These results suggest that the TIGIT pathway was a promising therapeutic target for septic patients with preexisting malignancy.

## Methods

### Mice.

Both male and female C57BL/6J mice aged between 8 weeks and 12 weeks at the start of the study were used. All mice were purchased from The Jackson Laboratory and maintained at Emory University Division of Animal Resources.

### Cancer model.

LLC1 cells were subcutaneously injected in the right inner thigh to induce murine carcinoma model. LLC1 was cultured in RPMI 1640 medium supplemented with 10% FBS, 1% glutamine, 1% penicillin-streptomycin, and 1% HEPES. Each mouse received 50,000 LLC1 cells suspended in 0.1 mL of PBS, and tumors were allowed to grow for 3 weeks. Mice were checked daily for tumor growth beginning from the 7th d after injection. Mice with tumor sizes between 1.5 cm and 1.9 cm were then subjected to either CLP or sham surgery 3 weeks after injection. Specifically, mice were euthanized regardless of time after tumor inoculation if the tumor reached 2.0 cm or if it had become necrotic. Mice were also monitored for weight loss and sacrificed if a greater than 25% loss of baseline body weight was observed. The presence of the LLC tumors in nonseptic mice did not cause difficulties in ambulating or the ability to reach food or water during the entire study period (up to 4 weeks).

### Sepsis model.

Sepsis was established through CLP. All procedures followed the recommendations of the international expert consensus initiative for the minimum quality threshold in preclinical sepsis studies (MQTiPSS) (41). Sham animals underwent laparotomy alone. Animals were given buprenorphine (0.1 mg/kg; Reckitt Benckiser Healthcare) for pain control prior to surgery and then PRN as determined by either veterinary staff or the investigator. Per a previously published work (42), a 1-cm incision was made in the midline of the abdomen and cecum was ligated 1 cm from the end with 4-0 surgical suture. The ligated cecum was perforated by a single through-and-through puncture with a 25-gauge needle and stool was extruded. After the incision was closed, mice were resuscitated with 1 mL of saline and monitored continuously as they awoke from anesthesia. Ceftriaxone (50 mg/kg; Sigma-Aldrich) and metronidazole (35 mg/kg; Sigma-Aldrich) were also administered immediately after CLP. Antibiotics were continued on a q12-h dosing schedule for 48 h postoperatively. For survival studies, eligible CA mice were subjected to CLP and treated with either anti-TIGIT or the same volume of PBS as septic control 1 h after abdominal closure. Mice were observed every 12 h during the 7 d and survival rates were recorded. For the endotoxic shock model, mice were injected (i.p.) with 80 μg poly(I:C) (LMW, InvivoGen) diluted in 100 μL of sterile PBS. Six h later, mice received 150 μg of *E*. *coli* O26:B6 LPS (L8274; Sigma) diluted in 200 μL of sterile PBS via i.p. injection. Mice were then treated with anti-TIGIT as below and followed for survival.

### Anti-TIGIT regimen.

Anti-mouse TIGIT monoclonal Ab (clone 1G9, Bio X Cell) was injected subcutaneously at a dose of 400 μg per mouse 1 h after CLP, and the injection was repeated 12 h later.

### Flow cytometry procedures and agents.

At the indicated time points after surgery, animals were sacrificed, and the spleens were collected and analyzed via flow cytometry as previously described (42). Specifically, the spleen tissue was passed through cell strainers with 70-mM pores (Falcon) to create single-cell suspensions. Cells were surface-stained with anti–CD4-Pacific blue, anti–CD62L-FITC, anti–CD69-PE, anti–NK1.1-APC, and anti–CD3-Alexa 700 (all from BD Biosciences); and anti–CD8-Pacific orange, anti–TIGIT-PE, anti–CD19-PerCP, anti–CD44-PerCP, anti–ICOS-PE-Cy7, anti–PD-1-APC-Cy7, and anti–CD25-APC-Cy7 (all from BioLegend).

For intranuclear staining, cells were stained with Abs against surface proteins and fixed and permeabilized with an eBioscience Foxp3 staining kit and then stained with anti–CTLA-PE and anti–Ki67-Alexa 700 (BioLegend), anti–Helios-FITC (BD Biosciences), and anti–Foxp3-APC (eBioscience).

For intracellular cytokine staining, 2 × 10^6^ splenocytes from each sample were plated in a 96-well plate. After centrifugation, cells were resuspended and incubated in RPMI 1640 culture medium containing 10% FBS (Mediatech), 2 mM L-glutamine, 0.01 M HEPES buffer, 100 mg/mL gentamicin (Mediatech), and 5 × 10^–5^ M 2-mercaptoethanol (Sigma-Aldrich). Then cells were stimulated with 30 ng/mL of PMA and 400 ng/mL of ionomycin (Sigma-Aldrich) in the presence of GolgiStop (BD Pharmingen) for 4 h at 37°C. After this, cells were surface stained with Abs against surface proteins, then cells were permeabilized by using fixation and permeabilization solution (eBioscience). We used anti–IL-2-FITC (BD Biosciences), anti–TNF-PE-Cy7 (BioLegend), and anti–IFN-γ-Alexa 700 (BD Biosciences) for intracellular cytokine staining. Samples were acquired and analyzed using an LSRII flow cytometer (BD Biosciences). A minimum of 3 x 10^6^ live cells were collected, and data were analyzed using FlowJo software (Tree Star).

### IHC.

Animals were sacrificed 48 h after CLP. Spleens were harvested and immediately fixed in 10% formalin. Paraffin-embedded tissues were washed in a serial alcohol preparation in a descending concentration. Slides were incubated with cleaved caspase-3 Ab diluted in PBS overnight at 4°C. After being rinsed 3 times with PBS, the slides were incubated with biotinylated anti-rabbit Ab diluted in PBS for 1 h at room temperature. Slides were developed with nickel-enhanced DAB substrate solution at for 5 min and then counterstained with hematoxylin. Cell death was quantified in the spleen by counting cells staining positive for active caspase-3, which stains brown, in 5 random, high-powered (×400) fields.

### Soluble cytokines and organ damage markers.

All mice were sacrificed 24 h after CLP and blood collected via cardiac puncture. Samples were centrifuged for 10 min at 4°C with 1000 *g*. Serum was procured from each sample for analysis. Soluble cytokine concentrations of IL-1β, IL-6, IL-10, MCP-1, TNF-α, and INF-γ were determined using the Bio-Plex 200 System with suspension array kits according to the manufacturer’s instructions (Bio-Rad). All samples were run in duplicate. Results were analyzed using the Bio-Plex Manager 3.0 Software. Levels were reported in pg/mL. Organ damage marker concentrations were obtained from serum by ELISA using the BioTek Synergy HT plate reader (BioTek). These markers included ALT (ABclonal), AST (Abcam), and creatinine (Arbor Assays). All kits were used according to the respective manufacturer’s instructions. Results were analyzed using Gen5 Image+ software (BioTek). Levels were reported in pg/dL and mg/dL.

### Statistics.

All data were analyzed using GraphPad Prism 7.0 software. Continuous variables were expressed as mean ± SD and were compared via 2-tailed Student’s *t* test or a Mann-Whitney *U* test given Gaussian distribution or not. Multigroup differences in flow cytometric data were evaluated using 2-way ANOVA and Tukey’s test. Survival data were analyzed by the log-rank test. *P* values less than 0.05 were considered significant.

### Study approval.

All experiments were approved by the Institutional Animal Care and Use Committee of Emory University (protocol: DAR-2003199-071418BN). Emory University IACUC guidelines regarding euthanasia of tumor-bearing mice were followed.

## Author contributions

WZ, JCA, KMR, CWC, JX, ZL, DBC, and SO performed experiments, WZ, JCA, CMC, and MLF designed experiments, analyzed and interpreted data, MLF and WZ wrote the paper, all authors edited the paper.

## Supplementary Material

Supplemental data

## Version 1 06/08/2021

Electronic publication
