# Supplementary material for: TIGIT modulates sepsis-induced immune dysregulation in mice with preexisting malignancy
Source: JCI Insight. 2021 Jun 8;6(11):e139823. doi: 10.1172/jci.insight.139823 (PMC8262279; doi:10.1172/jci.insight.139823)
Supplement: Supplemental data [file jciinsight-6-139823-s166.pdf]

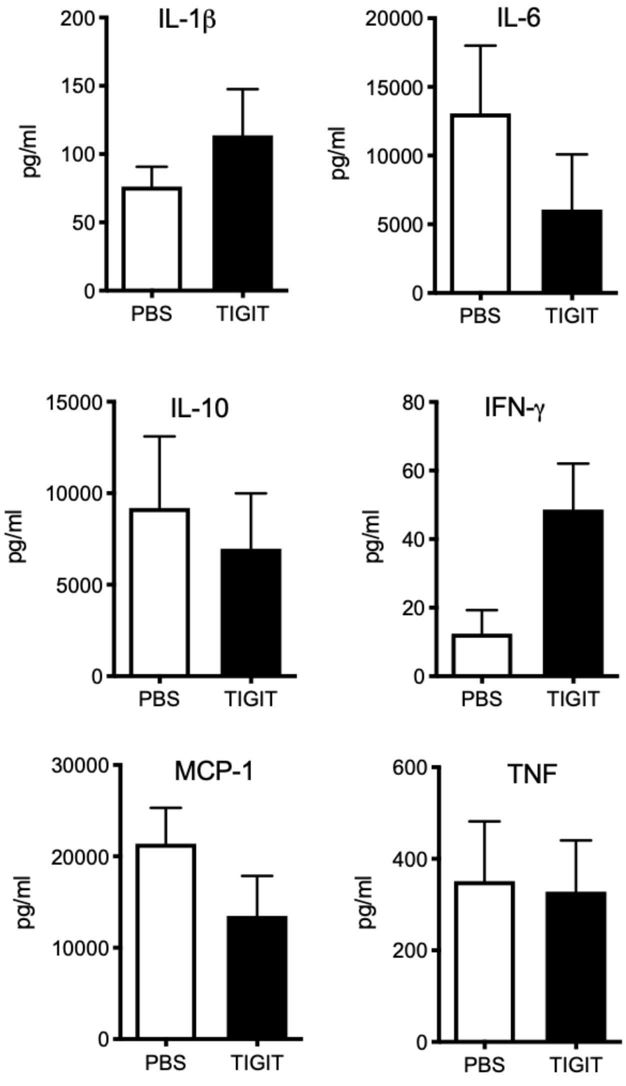

Figure S1. Anti-TIGIT treatment does not alter serum cytokines in cancer septic hosts.

Cancer mice were subjected to CLP and treated with anti-TIGIT mAb or PBS. Serum was collected at 48h post CLP and analyzed for the cytokines shown above.

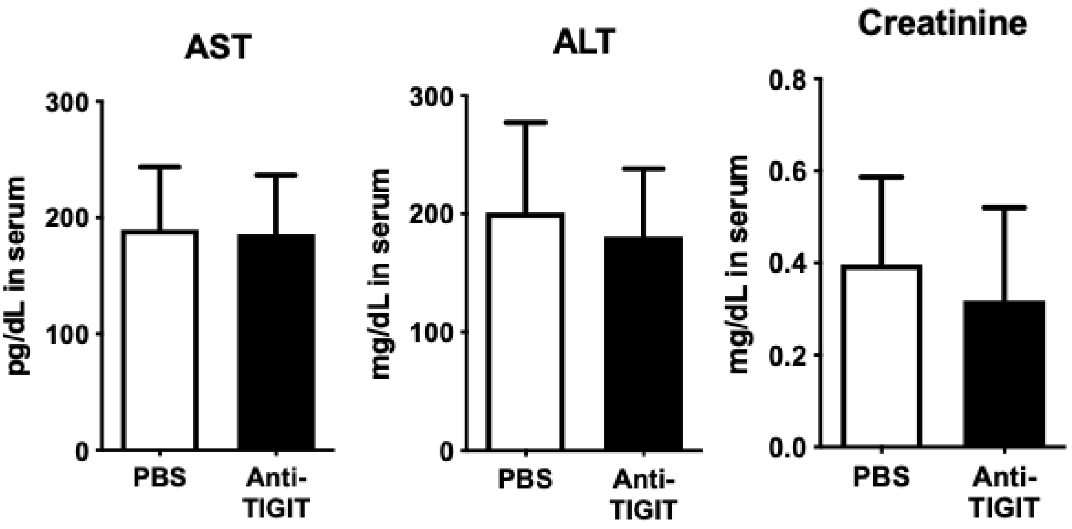

Figure S2. Anti-TIGIT treatment does not alter serum AST, ALT, or creatinine in cancer septic hosts. Cancer mice were subjected to CLP and treated with anti-TIGIT mAb or PBS. Serum was collected at 48h post CLP and analyzed for the markers of organ damage shown shown above.

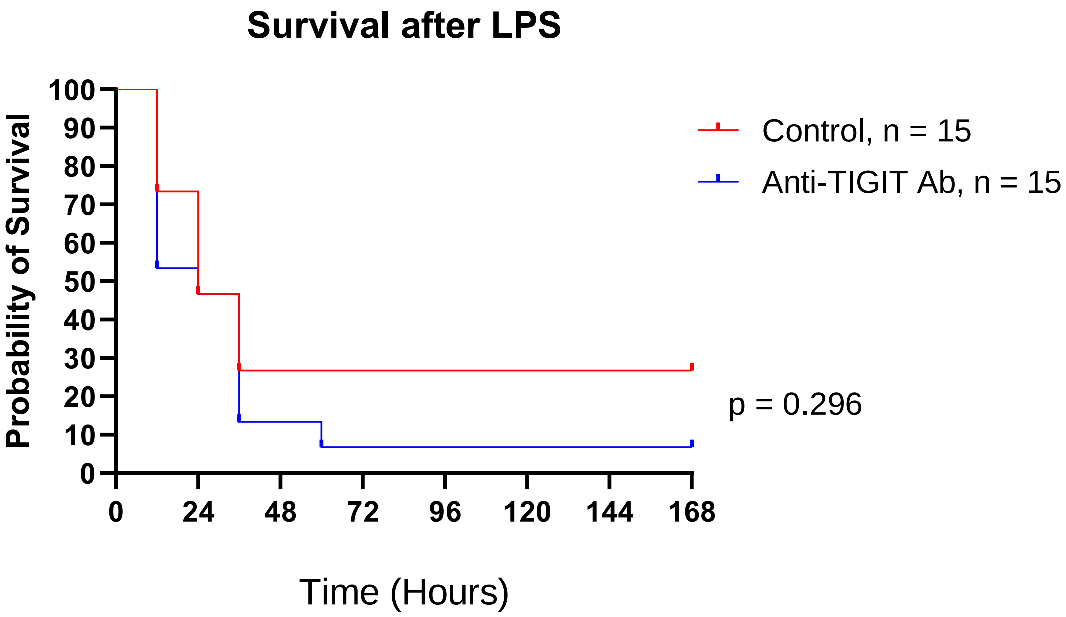

Figure S3. Anti-TIGIT does not impact mortality of cancer mice in a model of endotoxic shock. B6 mice were injected with LLC cells and tumors were allowed to develop for 30 days. Mice were injected with 80 µg poly(I:C) and six hours later, received 150 µg of *E. coli* O26:B6 LPS via intraperitoneal injection. Anti-mouse TIGIT monoclonal antibody was injected subcutaneously at a dose of 400 µg per mouse 1h after CLP, and the injection was repeated 12h later. Control animals received PBS injections. Animals were monitored for survival.

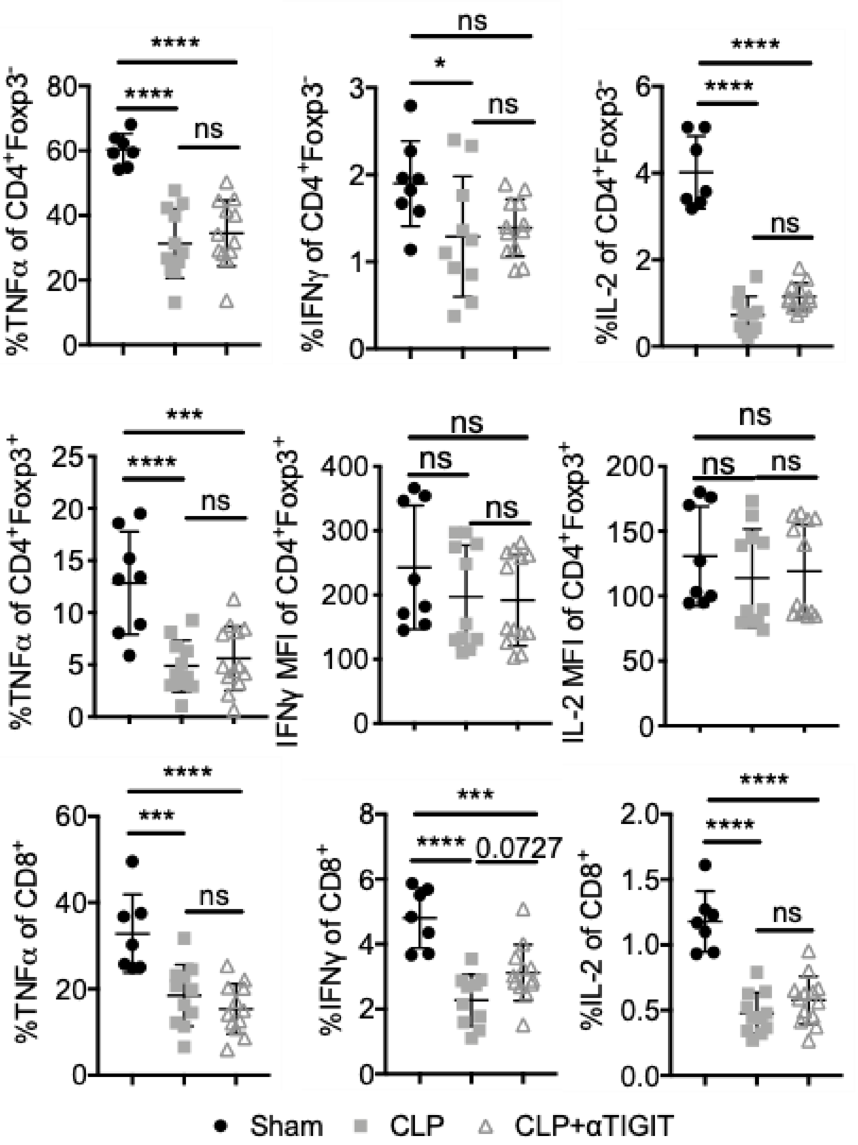

Figure S4. Anti-TIGIT treatment does not alter cytokine secretion in the CD4$^+$ and CD8$^+$ T cells of cancer septic hosts. Cancer mice were subjected to CLP (or sham surgery) and treated with anti-TIGIT mAb or PBS. Splenocytes from each group were stimulated with PMA and ionomycin at 37℃ for 4 hours. Intracellular staining of TNF, IFNγ and IL-2 was performed. Data were pooled from 2 independent experiments. Percentages of TNF$^+$, IFNγ$^+$ and IL-2$^+$ cells are displayed. *P $\leq$ 0.05, **P $\leq$ 0.01, ***P $\leq$ 0.001, ****P $\leq$ 0.0001.

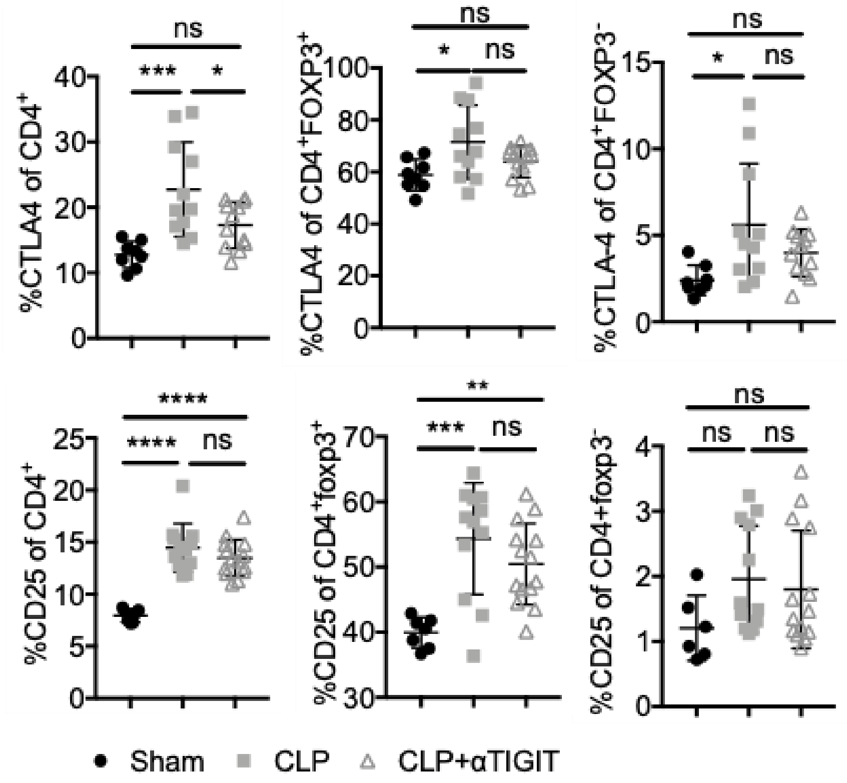

Figure S5. Anti-TIGIT treatment decreases CTLA-4 expression on bulk CD4$^+$ T cells in cancer septic mice. Cancer mice were subjected to CLP (or sham surgery) and treated with anti-TIGIT mAb or PBS. Two days after CLP, mice from each group were sacrificed and spleens were collected for CTLA-4 and CD25 staining. Summary data of CTLA4 and CD25 expression on total CD4$^+$ T cells, Treg cells and Tconv is displayed. *$P \leq 0.05$, **$P \leq 0.01$, ***$P \leq 0.001$, ****$P \leq 0.0001$.
